# Asbestos exposure and malignant mesothelioma of the tunica vaginalis testis: a systematic review and the experience of the Apulia (southern Italy) mesothelioma register

**DOI:** 10.1186/s12940-019-0512-4

**Published:** 2019-08-30

**Authors:** Luigi Vimercati, Domenica Cavone, Maria Celeste Delfino, Luigi De Maria, Antonio Caputi, Giovanni Maria Ferri, Gabriella Serio

**Affiliations:** 1 Interdisciplinary Department of Medicine (DIM), Unit of Occupational Medicine, Section Occupational Medicine. “B. Ramazzini”, University Aldo Moro of Bari Medical School, 11 G. Cesare Square, 70124 Bari, Italy; 2 Department of Emergency and Organ Transplantation (DETO), Pathology Division, University Aldo Moro of Bari Medical School, 11 G. Cesare Square, 70124 Bari, Italy

**Keywords:** Asbestos, Mesothelioma, Tunica vaginalis, Review, Apulia southern Italy, Mesothelioma register

## Abstract

**Background:**

Malignant mesothelioma of the tunica vaginalis testis (MMTVT) is a rare disease with a poor prognosis. The diagnosis and management of these lesions are often difficult for pathologists, surgeons, oncologists and occupational physicians. A preoperative diagnosis of malignancy is rarely made, and there is no established effective therapy except orchidectomy.

**Methods:**

A systematic literature review was conducted among the articles published in the English literature on primary MMTVT. Moreover four cases from the Apulia mesothelioma register are reported here.

**Results:**

Two hundred eighty-nine cases of MMTVT have been reported from 1943 to 2018. Overall asbestos exposure has been investigated only for 58% of all cases reported in this review, while in 41.8% this data are not available. Noteworthy is the fact that in many reports there is not an anamnestic reconstruction of any asbestos exposure. A history of direct occupational, environmental or familial asbestos exposure is found in 27.6% of the cases. The four cases from the Apulia mesothelioma register are all with ascertained occupational exposure to asbestos.

**Conclusions:**

The true incidence of asbestos exposure in MMTVT is underestimated because of insufficient information reported in older literature. To establish a broad consensus on the causal relationship between asbestos and MMTVT in the scientific community its necessary to analyze the same variables in the epidemiological studies. In general it should be recommended that a positive history of exposure to asbestos or to asbestos–containing materials are at risk for the development of a MMTVT and should be monitored.

## Background

Malignant mesothelioma (MM) is a rare tumour that can occur in the body cavities covered by mesothelium, i.e., the pleura, peritoneum, pericardium and testicular vaginal tunica [1], with benign and malignant variants. Among MM cases, a very small percentage (< 3%) [2] arise in the tunica vaginalis testis. Malignant mesothelioma of the testicular vaginal tunica (MMTVT) is very rare with potentially aggressive behaviour, and it can invade the testicular parenchyma, spermatic cord, epididymis and subcutaneous tissue of the penis; therefore, it has also been classified with the term paratesticular mesothelioma [3], rather than adenomatoid tumours, malignant adenomatoid tumours, mixed mesoblastic tumours or other various diagnoses, which is how it has been misinterpreted in the past [4–9]. The confusion over nomenclature was due to the difficulty of histological classification [10].

Over the years, three groups of mesothelial tumours have been identified, defined and classified: well differentiated papillary mesothelioma (WDPM); an emerging diagnostic category of papillary mesothelioma with borderline features or localized mesothelioma of low grade malignancy, also called mesothelioma of uncertain malignant potential (MUMP); and mesothelioma of low malignancy potential (MLMP) [11, 12], representing a morphological continuum between WDPM and malignant mesothelioma (MM) [11, 13].

As reported by Rankin (1956) [10] and by Kossow (1981) [14], the first two cases of mesothelioma of the genital tract were reported in 1912 by Naegeli [15] and in 1916 by Sakaguchi [16], followed by Thompson (1936), Evans (1943), Golden and Ash (1945), Lee (1950), Bailey (1955) and Barbera (1957) [4, 17–21], although the last was actually classified as benign papillary mesothelioma (WDPM). These cases were described by various names until 1970, when Marcus and Lynn [22] demonstrated by electron microscopy that there were no differences between so-called adenomatoid tumours and malignant mesothelioma [23].

To date, the WHO(world health organization) classification of tumours of the urinary system and male genital organs [24], in the classification of tumours of the testis and paratesticular tissue, has reported MM and the WDPM, noting that the latter “*may have a progression to malignant mesothelioma if the lesions are not completely excised”.*

This review points out only case series and case reports of primary MM of the tunica vaginalis testis. We conducted a comprehensive review from Medline (National Library of Medicine database) and a PubMed database search of the English medical literature and on the references lists of published articles. Nevertheless, the data are often incomplete or not comparable due to the long period covered by the scientific literature examined (1943-2018) and the relative evolution of diagnostic techniques and classifications of mesotheliomas, as well as knowledge about the risk factors related to the onset of the disease [5, 21, 25, 26]. Similarly, despite the rarity of this disease, all of the various reviews reported might not indicate the true incidence because of the relatively recent agreement about the definition of the clinicopathologic entity. In addition, we report four cases from the Apulia (Southern Italy) mesothelioma register.

## Methods

A PubMed computerized search was performed using the following keywords: mesothelioma tunica vaginalis testis (127 articles), testicular (276 articles), paratesticular (50 articles), testis (179 articles), and scrotum (46 articles); and it was filtered for human patients and English language. The English literature search without time limits, from 1943 to 2018, the cut-off date was December 15, 2018, and were identified a total of more than 276 previously published scientific articles on MMTVT (MEDLINE-PUBMED National Library of Medicine, National Center for Biotechnology Information; available from URL: http://www.ncbi.nlm.nih.gov/pubmed).

We undertook a review using the following criteria: we excluded articles with the diagnosis of benign mesothelioma as stated by the authors on the basis of histopathological findings and cases of adenomatoid tumours and other benign tumours and WDPM [27], although some authors, such as Grove (1989) [28], suggested that these tumours should always be considered “borderline malignancy”. Similarly, cases with doubtful primary tumour origin or with concomitant pleural or peritoneal disease were excluded.

Using the above criteria, the review of the literature to date revealed 289 previously described cases in 165 published articles from PubMed and from the reference lists of the available publications in the English literature, which we considered bona fide malignant MTVT(Table 1) [4, 5, 9, 11–14, 23, 28–184].

**Table 1:** Summary features MMTVT cases (1943-2018)

N°	Year	Author (Reference)	N° Cases	Age Years	Asbestos Exposure (latency in years)	Follow Up Months	Side-Laterality	Histologic Subtype	Recurrence Months	Clinical Presentation Onset
1	1943	Evans [4]	2	66	NA	2	LEFT	NA	NA	MASS
				53	NA	NA	LEFT	NA	NA	SMALL NODULE PAINLESS
2	1945	Robinson [28]	2	30	NA	NA	NA	NA	NA	NA
				28	NA	NA	NA	NA	NA	NA
3	1947	Patterson [29]	1	NA	NA	NA	NA	NA	NA	NA
4	1949	Foote [30]	1	NA	NA	dead	NA	NA	metastasis	NA
5	1949	Fajers [5]	5	27	NA	NA	RIGHT	NA	NA	NA
				35	NA	NA	LEFT	NA	NA	NA
				50	NA	NA	LEFT	NA	NA	NA
				45	NA	NA	RIGHT	NA	NA	NA
				58	NA	Na	RIGHT	NA	NA	NA
6	1958	Reynolds [31]	1	45	NO	6 alive	RIGHT	papillary epithelioid	NA	LARGE MASS HYDROCELE
7	1968	Kozlowski [32]	1	63	NA	NA	spermatic cord	biphasic	NA	MASS
8	1968	Abell [9]	2	78	NA	45 dead	NA	biphasic	metastasis	NA
				70	NA	16 dead	NA	biphasic	metastasis	NA
9	1969	Kasdon [33]	2	58	NA	36 dead	RIGHT	papillary epithelioid	12	HYDROCELE
				72	NA	36 recurrence	RIGHT	papillary epithelioId	5	HYDROCELE
10	1969	Arlen [34]	1	40	NA	216	LEFT spermatic cord	NA	metastasis	MASS
11	1973	Johnson [35]	1	23	NA	3	RIGHT	papillary epithelioid	3 alive	SWELLING AND MILD DISCOMFORT
12	1975	Fishelovitch [36]	1	60	NA	12 alive	LEFT	papillary epithelioid	NO	SWELLING, HYDROCELE
13	1976	Fligiel [37]	1	68	pipe insulator for 40 years (pleural plaque)	20 dead	RIGHT	papillary epithelioid	NA	PAIN AND SWELLING
14	1976	Pugh [38]	2	NA	NO	48 alive	NA	papillary epithelioid	NO	HYDROCELE
				NA	NO	84	NA	papillary epithelioid	84 recurrence	HYDROCELE
15	1976	Pizzolato [39]	1	57	Sugar raffinery worker	41 dead	RIGHT	papillary epithelioid	12 recurrence	URETHRAL STRUCTURE, SECONDARY URINARY EXTRAVASION WITH URETHRITIS AND RECURRENT INGUINAL HERNIA
16	1977	Eimoto [40]	1	35	NO	2 dead	LEFT	fibrous	NA	SWELLING
17	1977	Tuttle [41]	1	37	NA	NA	RIGHT spermatic cord	fibrous	NA	MASS
18	1978	Sinha [42]	1	65	NA	24 alive	RIGHT	papillary epithelioid	NA	SWELLING
19	1978	Jaffe [43]	1	77	NO	12 dead	LEFT	papillary epithelioid	Local recurrence	SWELLING
20	1981	Benisch [44]	1	64	NO	8	SCROTUM NA	fibrous	NO	MASS
21	1981	Kossow [14]	1	50	NA	24 NED	RIGHT	papillary epithelioid	NO	MASS
22	1981	Blitzer [45]	1	74	NA	30	LEFT spermatic cord	NA	NO	PAINLESS, MASS
23	1982	Japko [46]	1	30	Insulator for 8 years	6 NED	RIGHT	Biphasic	NO	SWELLING
24	1982	Chen [47]	1	64	NA	30 dead	RIGHT	Biphasic	24 recurrence	SWELLING
25	1982	Hollands [48]	1	63	NA	24	RIGHT TESTIS	Biphasic	12 recurrence	HYDROCELE, SWELLING
26	1982	Slaysman [49]	1	20	NA Maffucci Syndrome	NA	bilateral	papillary epithelioid	Recurrence bilateral	SWELLING
27	1983	Zidar [50]	1	63	NA	32	NA	papillary epithelioid	NA	NA
28	1983	Mc Donald [51]	2	21	NA	24	RIGHT HEMI-SCROTUM bilateral	papillary epithelioid	24 recurrence	PAINFUL GRADUAL ENLARGMENT
				29	NA	20 NED	RIGHT HEMI-SCROTUM	papillary epithelioid	NO 20	PAINFUL GRADUAL ENLARGMENT
29	1983	Van Der Rhee [52]	1	86	NO	36 dead	LEFT HEMI-SCROTUM	papillary epithelioid	12 recurrence	HAEMATOSCROTUM
30	1984	Antman [53]	6	58	pipefitter	60	LEFT INGUINAL	papillary solid polypoid tissue	60 metastasis	HYDROCELE, INGUINAL HERNIA
				73	shipyard plumber for 25 years	33 dead	RIGHT	papillary epithelioid	12 metastasis	HYDROCELE
				23	NA	180	LEFT	NA	180 metastasis	SLOWLY ENLARGEMENT
				63	machinist for 20 years	48 dead	LEFT	papillary epithelioid	4 metastasis	MASS
				52	NO	24 dead	RIGHT	papillary epithelioid	14 metastasis	MASS
				43	construction worker for 16 years	48 NED	RIGHT	papillary epithelioid	NO	EPIDIDIMITE
31	1984	Yamanishi [54]	1	34	NA	6	RIGHT	biphasic	NO	MASS
32	1984	Khan [55]	1	42	NO	9 dead	RIGHT epididymis	biphasic	6 metastasis	PAINFULL, SWELLING
33	1985	Vakalikos [56]	1	26	NA	12 NED	RIGHT	papillary pseudotu-bular	NO 12	SWELLING INGUINAL
34	1985	Ehya [57]	1	63	NA	52	LEFT	papillary epithelioid	50 metastasis	HYDROCELE
35	1986	Karunaharan [58]	1	40	plastic fenolica worker for 20 years	14 dead	RIGHT	glandular structure papillary epithelioid	9 metastasis 12 local recurrence	IRREGULAR MASS
36	1986	Petersen [59]	1	51	NA	30	NA	NA	30 alive recurrence	NA
37	1987	Cartwright [60]	1	49	NA	4	RIGHT	papillary epithelioid	24 metastasis	HYDROCELE
38	1987	Fitzmaurice [61]	1	72	NA	18 NED	LEFT	papillary epithelioid	NO 18	SWELLING
39	1988	Linn [62]	1	20	NA	NA	LEFT	papillary epithelioid	NA	PAIN, SWELLING
40	1988	Prescott [63]	1	61	Pleural plaques	21 dead	LEFT	Biphasic papillary, pseudo-glandular component	8 Local recurrence metastasis	HYDROCELE
41	1988	Velasco [64]	1	14	NA	24	LEFT	papillary epithelioid	NO 24	ABDOMINAL MASS
42	1989	Tyagi [65]	1	79	Shipyard worker	24 dead	LEFT	papillary epithelioid	metastasis	SWELLING
43	1989	Grove [27]		66	Carpenter for 10 years	42	RIGHT	papillary epithelioid	24 metastasis 42 alive local recurrence	SWELLING HYDROCELE
				79	NO	60 dead	RIGHT	epithelial papillary	60 Prostatic metastasis	MASS
				58	NO	108 NED	LEFT	Tubule papillary	108 NED	HYDROCELE
44	1990	Kamiya [66]	1	32	NO	5	LEFT	papillary	NO 5	ELASTIC AND INDOLENT TUMOR
45	1990	Smith [67]	1	57	NO	alive	LEFT	papillary epithelioid	48 Local recurrence metastasis	SWELLING
46	1990	Carp [23]	1	54	NO	64 dead	LEFT	papillary epithelioid	38 Local recurrence metastasis	MASS
47	1991	Kuwabara [68]	1	60	NO	65 dead	RIGHT	Biphasic	60 metastasis	SWELLING
48	1992	Pfister [69]	1	7	NA	16	LEFT	papillary epithelioid	NO 16	SWELLING
49	1992	Adler [70]	1	62	occupational	12	RIGHT	NA	NO 12	PAINFUL ENLARGEMENT
50	1992	Serio(7 [71]	1	69	railway cleaner for 10 years	10	LEFT	tubulo-papillary	NO 10	SWELLING
51	1992	Noble [72]	1	62	NA	NA	LEFT	papillary epithelioid	NA	SWELLING, HYDROCELE
52	1992	Fields [73]	1	91	Steel industry worker indirect exposure	NA	NA	Biphasic	NA	SWELLING
53	1994	Moch [74]	1	80	NO	25 NED	RIGHT	papillary epithelioid	NO	PAINLESS SWELLING
54	1994	Saw [75]	1	63	Occupational for 7 years [20]	6	LEFT	Biphasic	NO 6	HYDROCELE
55	1994	Reynard [76]	1	76	NA	NA	RIGHT	tubulo-glandular	1 recurrence	PAINLESS SWELLING
56	1994	Wenger [77]	1	25	NA	NA	RIGHT	NA	NA	PAINFUL MASS
57	1994	Watanabe [78]	1	67	Insulator for 17 years asbestosis	10 dead	LEFT	biphasic	multifocal	NA
58	1995	Amin [79]	1	59	NO	189	RIGHT	papillary epithelioid	NO 188	PAINLESS SWELLING
59	1995	Magoha [80]	1	NA	NA	NA	NA	fibrous	NA	NA
60	1995	Huncharek [81]	1	45	insulator Electrical power plant	144	RIGHT	epithelial	NO	PAINLESS MASS
61	1995	Umekawa [82]	1	67	NO	8 dead	RIGHT	epithelial	6 metastasis	SWELLING
62	1995	Eden [83]	2	62	NO	6	LEFT	NA	NO 6	HYDROCELE
				76	Chemist for 10 years	27 alive	LEFT	epithelial	6 recurrence	HYDROCELE
63	1995	Joseph [84]	1	26	NA	NA	LEFT	NA	NA	TWO PINK TO-PURPLE NODULES
64	1995	Lopez [85]	1	47	NA	36	NA	papillary epithelioid	NO 36	HYDROCELE
65	1995	Jones [86]	11	75	NA	Lost follow up	NA	epithelial	NA	HYDROCELE
				12	NA	12 ned	NA	epithelial	NO 12	HYDROCELE
				39	NO	3	NA	epithelial	NA	PARATESTICULAR MASS
				50	NO	24	NA	epithelial	24 local recurrence	HYDROCELE
				41	NO	3	NA	biphasic	NA	PARATESTICULAR MASS
				65	NA	180	NA	biphasic	180 metastasis	PARATESTICULAR MASS
				76	pipe fitter for 10 years	48 dead	NA	biphasic	NA	HYDROCELE
				58	NA	36 dead	NA	biphasic	NA	HYDROCELE
				67	NO	3	NA	biphasic	NA	PARATESTICULAR MASS
				70	NA	48 dead	NA	epithelial	NA	HYDROCELE
				42	NA	24	NA	epithelial	Alive with disease	HYDROCELE
66	1996	Ahmed [87]	1	80	dock worker for 10 years	24 dead	LEFT	papillary epithelioid	3 local recurrence	HYDROCELE
67	1996	Ascoli [88]	1	55	Insulator	6	RIGHT	biphasic	NA	SWELLING
68	1996	Mathew [89]	2	70	NA	3 dead	LEFT	NA	spinal metastasis	SWELLING
				58	NA	2 dead	RIGHT	NA	spinal metastasis	ENLARGMENT
69	1997	Berti [90]	1	75	NO	15	LEFT	papillary epithelioid	NO 15	HYDROCELE
70	1997	Agapitos [91]	2	60	NA	20	LEFT	biphasic	NA	SWELLING
				84	NA	10	LEFT	biphasic	NO 10	HYDROCELE
71	1997	Khan [92]	1	6	NA	24	BILATERAL	papillary epithelioid	NO 24	HYDROCELE
72	1998	Gupta SC [93]	1	36	NO	10	RIGHT	papillary epithelioid	1,5 metastasis	HYDROCELE
73	1998	Lee [94]	2	45	NA	4	RIGHT	NA	4 metastasis	HYDROCELE
				66	NA	6	LEFT	papillary epithelioid	NO 6	HYDROCELE
74	1998	Plas [95]	1	14	NO	12	RIGHT	papillary epithelioid	NO 12	ENLARGEMENT
75	1999	Kanazawa [96]	1	38	maintenance air conditioning system for 20 years	156	BILATERAL	epithelial	36 local recurrence	INGUINAL HERNIA
76	1999	Harmse [97]	1	70	NO	120	RIGHT	epithelial	NA	MASS
77	1999	Gupta NP [98]	2	69	NA	18 dead	RIGHT	biphasic	metastasis	SWELLING
				51	NA	5 dead	LEFT	biphasic	metastasis	ENLARGEMENT
78	2000	Fujisaki [99]	1	32	NO	36	RIGHT	epithelial	NO 36	SWELLING
79	2000	Poggi [100]	1	47	NA	8	RIGHT	epithelial	NA	PARATESTICULAR MASS
80	2000	Attanoos [13]	3	71	dockyard crane driver for 20 years (ASBESTOS BODIES)	NA	RIGHT	biphasic	NA	HYDROCELE
				77	NO	50	LEFT	epithelial	NA	MASS
				33	NO	37	LEFT	epithelial	NA	MASS
81	2000	Ferri [101]	1	64	NA	36	NA	epithelial	NA	OSTRUZIONE CERVICO-URETRALE
82	2001	Wolanske [102]	1	71	NO	3	RIGHT	NA	NO 3	NODLE
83	2001	Sebbag [103]	2	34	NO	62 alive	LEFT	epithelial	NO 6O	INGUINAL MASS
				19	NO	24 dead	LEFT	epithelial	11 recurrence	SCROTAL MASS
84	2001	Gurdal [104]	1	67	NO	30	RIGHT	epithelial	24 recurrence	HYDROCELE RECIDIVANTE
85	2002	Abe [105]	1	81	NO	12 dead	LEFT	epithelial	7 metastasis	HYDROCELE
86	2002	Bruno [106]	1	85	NO	NA	RIGHT HEMI-SCROTUM	epithelial	NA	SWELLING
87	2002	Iczkowski [107]	1	71	NO	26 dead	LEFT HEMI-SCROTUM	epithelial	19 liver metastasis	PAINFULL SWELLING
88	2003	Black [108]	1	67	NA	36 dead	RIGHT	epithelial	3 recurrence	HYDROCELE
89	2003	Garcia de Jalon [109]	1	78	carpenter	3	RIGHT	tubulo-papillary	3 metastasis	INCREASE IN THE VOLUME THE TESTIS
90	2004	Pelzer [110]	1	21	NO	24	BILATERAL	epithelial	NA	RECURRENT PAIN
91	2004	Sawada [111]	1	48	NO	72	RIGHT	biphasic	NO 72	SWELLING
92	2004	Mishra [112]	1	75	NA	NA	NA	NA	NA	NA
93	2004	Shimada [113]	1	64	NO	18	RIGHT	biphasic80% sarco-matoid	NO	SWELLING
94	2005	Wang [114]	1	81	NO	NA	RIGHT	tubulo-papillary	NA	SCROTAL MASS
95	2005	Gorini [115]	2	67	maintenance of locomotives for 30 years [42]	24	LEFT	epithelial	NO 24	MASS
				80	maintenance of tractors for 6 years [67]	24	RIGHT	biphasic	NA	SWELLING
96	2005	Spiess [116] (**no individual data)**	5	57-83	4	5-68 (4 dead 1 disease free survival 68)	NA	NA	Metastasis in 4 cases	NA
97	2006	Van Apeldoorn [117]	1	83	NO but with pleural thickening at CT	1 dead	RIGHT	epithelial	liver metastasis	SCROTAL ENLARGEMENT Chyluria
98	2006	Schure [118]	3	45	NO	48	LEFT	NA	NO 48	SWELLING
				35	NO	4 dead	LEFT	NA	2 metastasis	MASS INGUINO-SCROTAL
				26	NO	18	LEFT	NA	NO 18	INGUINAL MASS
99	2006	Winstanley [119]	18	54	NO	12 dead	RIGHT	NA	NA	FOLLOWING A FALL
				56	Dockyard worker	60	NA	NA	60 metastasis	SOVRAPUBIC MASS
				59	NO	24 dead	LEFT	NA	NA	RECURRENT HYDROCELE
				52	NO	48	LEFT	NA	NO 48	RECURRENT HYDROCELE
				49	NA	24	NA	NA	NO 24	HYDROCELE
				79	NA	1	LEFT	NA	NO 1	HYDROCELE
				70	NA	12 dead	RIGHT	NA	NA	BLOOD STAINED HYDROCELE
				62	NA	24 dead	LEFT	NA	NO	RECURRENT HYDROCELE
				45	NA	6	LEFT	NA	NO 6	SWELLING TESTICULAR
				65	NA	60	RIGHT	NA	NO 60	HYDROCELE
				75	NO	36	LEFT	NA	NA	HYDROCELE
				73	NO	12 dead	LEFT	NA	12 metastasis	TESTICULAR SWELLING
				45	NA	72	NA	NA	NO 72	NA
				58	NO	36	LEFT	NA	NO 36	HYDROCELE HISTORY E YEARS
				NA	NA	NA	NA	NA	NA	NA
				NA	NA	NA	NA	NA	NA	NA
				NA	NA	NA	NA	NA	NA	NA
				NA	NO	NA	NA	NA	NA	NA
100	2007	Al Qahtani [120]	1	39	NA	84	LEFT	NA	NO 84	HYDROCELE
101	2007	Liguori [121]	1	68	NA	70	LEFT	epithelial	24 recurrence	INGUINAL MASS
102	2007	Guney [122]	1	45	NO	3	RIGHT	papillary	NO 3	TESTICULAR MASS
103	2008	Boyum [123]	1	60	NO	23	LEFT	biphasic	NA	SCROTAL SWELLING RECURRENT EPIDIDYMITIS
104	2008	Candura [124]	1	38	petrochemical worker for 16 years	15	RIGHT	epithelial	NO 15	HYDROCELE
105	2008	Mathur [125]	2	65	Farmer	NA	LEFT	papillary	NA	SWELLING
				60	NA	NA	RIGHT	epithelial	NA	SWELLING
106	2008	Ikegami [126]	1	67	Painting worker	26 dead	RIGHT	epithelial	24 liver metastasis	PAINLESS SWELLING
107	2008	Barui [127]	1	42	NO	NA	RIGHT	tubulo-papillary	NA	SCROTAL MASS
108	2008	Goel [128]	1	65	Farmer	72	LEFT	epithelial	NA	PAINLESS SWELLING
109	2009	Al Salam [129]	1	83	NO	NA	LEFT	epithelial	NA	SCROTAL SWELLING
110	2009	Baccheta [130]	1	63	NO	30 dead	RIGHT	epithelial	NA	NA
111	2009	Chen [131]	1	67	Occupational [40]	7	RIGHT	biphasic	NO 7	RIGHT HYDROCELE AND LONG-STANDING BILATERAL HYDROCELE
112	2009	De Lima [132]	1	15	NO	12	RIGHT	epithelial		PAINLESS INCREASE IN SCROTUM VOLUME
113	2010	Brimo MUMP [11]	8	43	NA	108 alive	NA	papillary tubulo papillary	108 NED	HYDROCELE
				49	NA	24 alive	NA	papillary tubulo papillary	24 NED	HYDROCELE
				73	NA	8 alive	NA	papillary tubulo papillary	8 NED	HYDROCELE
				34	NA	36 alive	NA	papillary tubulo papillary	36 NED	SCROTAL MASS
				61	NA	60 dead	NA	papillary tubulo papillary	18 NED	HYDROCELE
				53	NA	564 dead	NA	papillary tubulo papillary	564	HYDROCELE
				57	NA	NA	NA	papillary tubulo papillary	NA	HYDROCELE
				50	NA	NA	NA	papillary tubulo papillary	NA	HYDROCELE
114	2010	Aggarwal [133]	1	75	NA	76 dead	LEFT	NA	30 recurrence	SCROTAL ENLARGEMENT
115	2010	Bisceglia [134]	1	74	NO	101	RIGHT	tubulo-papillary	24 recurrence	TESTICULAR PAIN
116	2010	Klaassen [135]	1	37	NO	6	LEFT	papillary epitheliode	6 NO	MASS
117	2011	Trpkov [12]MUMP	1	57	NO	72 NED	NA	papillary epitheliode	NO	HYDROCELE
118	2011	Gupta R [136]	1	80	NA	NA	RIGHT	tubulo- papillary	NA	SWELLING OF 3 YEARS DURATION
119	2011	Park [137]	1	65	Foundry worker for 4 years	6 dead	LEFT	papillary	3 recurrence	PALPABLE MASS
120	2011	Grey Venyo [138]	1	69	NO	NA	LEFT	epithelial	2 recurrence	SWELLING
121	2011	Bass [139]	1	64	worked on a naval vessel	44 alive	LEFT	papillary	20 recurrence	SCROTAL SWELLING
122	2012	Ahmed [140]	1	78	NO	6	RIGHT	epithelioid	NO 6	PAINFUL SWELLING
123	2012	Whan Doo [141]	1	36	NO	1	RIGHT	NA	NO 1	PAINLESS SWELLING
124	2012	Abdelrahman [142]	1	54	Farmer	NA	RIGHT	biphasic	NA	SWELLING
125	2012	Esen [143]	1	38	NO	26	LEFT	epitheloid	NO 26	PAIN AND SWELLING
126	2012	Bo Hai [144]	6	26	NO	24	LEFT spermatic cord	epithelial	NO 24	SPERMATIC CORD MASS
				67	NO	24	LEFT	epithelial	Local recurrence	SCROTAL MASS, BILATERAL HYDROCELE
				57	NO	24	RIGHT spermatic cord	epithelial	Local recurrence	MASS
				46	YES	24 dead	LEFT	epithelial	DOD	ACUTE APPENDIX, TESTIS PAIN
				78	NO	24	LEFT	epithelial	Local recurrence	SCROTAL MASS, BILATERAL HYDROCELE
				76	YES	24 dead	LEFT	epithelial	DOD	SCROTAL MASS
127	2012	Priester [145]	1	71	NA	24 dead	RIGHT	epithelial	17 recurrence	HYDROCELE
128	2012	Heng Yen [146]	1	53	NO	36	LEFT	tubulopapillary	NO 36	RECURRENT EPIDIDYMITIS, HYDROCELE
129	2012	Mrinakova [147]	1	20	environmental	41	LEFT TESTIS	papillary	NO 41	PAINLESS HYDROCELE
130	2012	Mensi [148]	13	72	NA	8	RIGHT	epithelial	NA	HYDROCELE AND ENLARGEMENT
				73	Familial for 4 years	44	LEFT	biphasic	NA	HYDROCELE
				76	Occupational maintenace worker for 32 years	9	LEFT	epithelial	NA	SCROTAL HERNIA
				80	Household for 11 years	18	RIGHT	biphasic	NA	HYDROCELE
				60	NA	15	RIGHT	epithelial	NA	INGUINAL-SCROTAL HERNIA
				82	Occupational spinner for 32 years	25	LEFT	sarcomatous	NA	TESTICULAR MASS
				38	Occupational maintenace worker for 16 years	33	RIGHT	epithelial	NA	TESTICULAR PAIN AND SPERMATIC CORD TORSION
				69	NA	52	LEFT	desmoplastic	NA	HYDROCELE
				85	NA	14	LEFT	poorly differentation	NA	HYDROCELE
				69	NA	39	LEFT	sarcomatous	NA	TESTICULAR MASS
				76	Occupatonal textile worker for 11 years	42	RIGHT	epithelial	NA	HYDROCELE
				77	Occupational bricklayer for 24 years	8	LEFT	epithelial	NA	HYDROCELE
				74	Occupatonal bricklayer for 28 years	6	LEFT	epithelial	NA	TESTICULAR MASS, HYDROCELE
131	2012	Vijayan [149]	1	89	Familial (son asbestosis)	3 dead	LEFT	papillary	1 recurrence	SWELLING
132	2012	Shelton [150]	1	NA	NA	NA	NA	tubulo papillary	NA	NA
133	2012	Gemba **no individual data** [151]	5	na	3 /5 (construction, shipbuilding, steel production)	NA	NA	NA	NA	NA
134	2013	Busto Martin [152]	1	61	NO	120	RIGHT	biphasic	NO 120	INCREASE OF RIGHT SCROTUM SIZE WITH PAIN
135	2013	Gkentzis [153]	1	55	NA	NA	LEFT	epitheliod	24 recurrence	MASS PALPABLE
136	2013	Weng [154]	1	28	NO	12	LEFT	tubulo papillary	NO 12	SCROTAL TENDERNESS AND SWELLING
137	2013	Meng [155]	1	45	NO	6 alive NED	LEFT	epitheliod	NO	MASS
138	2013	Rajan [156]	1	18	NA	14 dead	LEFT	papillary,multycistic	10 metastasis	SCROTAL PAIN AND SWELLLING
139	2013	MeisenKothen [157]	9	60	Occupational asbestos cement pipe and domestic for 10 years [53]	15 dead	RIGHT	NA	15 recurrence	NA
				70	Familial domestic occupational mechanic for 30 years [64]	46 alive	RIGHT	epithelioid	NO 46	NA
				59	Domestic occupational US navy railroad [48]	71 alive	RIGHT	epithelioid	NO 71	MASS
				44	Occupational mining worker for 24 years [26]	14 dead	RIGHT Spermatic cord	epithelioid	14 DOD	NA
				74	occupational shipping industry for 30 years [58]	54 dead	RIGHT	biphasic	24 recurrence	INGUINAL MASS
				63	Occupational automobile manufacturing for 8 years [48]	1 dead	RIGHT	ephitelioid	1 metastasis	NA
				51	Hobby and occupational asbestos cement pipe for 7 years [41]	54 alive	LEFT	epithelioid	NO 54	HYDROCELE
				51	Occupational petrochemical plant worker and hobby for [31]	43 alive	RIGHT	NA	NO 43	NA
				65	Occupational mechanic and hobby for 23 years [49]	39	LEFT	epithelioid	NO 39	NA
140	2014	Lin Nei Hsu [158]	1	76	50 years house environmental residential	8	RIGHT	biphasic	NO	SWELLING
141	2014	Gomes da Fonseca [159]	1	62	NO	5 dead	LEFT	epithelioid	3 metastasis	ENLARGEMENT
142	2014	Stradella [160]	1	51	Possible occupational	NA	RIGHT	biphasic	NA	HYDROCELE
143	2014	Yang [161]	1	68	Farmer	6	RIGHT	epithelioid	NO	PAINFUL
144	2015	Akin [162]	1	49	NO	48	LEFT	papillary	NA	TESTICULAR MASS
145	2015	Bandyopadhyay [163]	1	40	Farmer	NA	NA	papillary	NA	SCROTAL SWELLING
146	2015	Jankovichova [164]	1	67	Environmental residential roof eternit and occupational lorry driver construction material	44	LEFT	epithelioid	14 local recurrence	HYDROCELE
147	2015	Silverio [165]	1	82	NA	NA	NA	NA	NA	NA
148	2015	D’Antonio [166]	1	80	Occupational railway workers	12	RIGHT spermatic cord	tubulo- papillarymm	NO 12	PAINLESS MASS
149	2015	Segura [167] Gonzales	1	58	NO	6	LEFT	epithelioid	NO 6	SWELLING
150	2015	Alesawi [168]	1	69	NO	12	RIGHT	tubulo- papillary	NO 12	HYDROCELE
151	2016	Hispan [169]	1	93	Occupational aluminum factory for 40 years	NA	LEFT	tubulo- papillary	NA cutaneous metastasis	MUTIPLE NODULES
152	2016	Mrinakova [170]	2	67	Occupational environmental	62	LEFT	tubulo- papillary	24 recurrence	HYDROCELE
				20	environmental	91	LEFT	epithelioid	NO 91	HYDROCELE
153	2016	Ahmed [171]	1	45	Occupaional truck driver	NA	spermatic cord	biphasic	NA	SWELLING
154	2016	Andresen [172]	1	60	Occupational	27	RIGHT	NA	24 recurrence	SWELLING hydrocele
155	2016	Serio [173]	2	77	Occupational machines ship	44 dead	LEFT	epithelioid	26 recurrence	SWELLING
				82	NO	63 dead	LEFT	epithelioid	53 recurrence	hydrocele
156	2016	Bertolotto [174]	7	64	NA	NA	RIGHT	epithelioid	NA	SCROTAL ENLARGEMENT
				60	NA	66	LEFT	epithelioid	NO 66	MASS
				65	NA	132 dead	LEFT	epithelioid	NA	SCROTAL ENLARGEMENT
				70	NA	24 dead	RIGHT	epithelioid	NA	SCROTAL ENLARGEMENT
				82	NA	6	RIGHT	epithelioid	NO 6	PALPABLE MASS
				63	NA	NA	BILATERAL	NA	NA	SCROTAL ENLARGEMENT
				75	NA	NA	RIGHT	NA	NA	PALPABLE MASS
157	2017	Zhang [175]	1	50	NA	24	LEFT	biphasic	NO 24	PAINLESS ENLARGEMENT
158	2017	Arda [176]	1	84	NA	NA	LEFT	epithelioid	NO	SCROTAL SWELLING
159	2017	Recabal [177] **No individual data**	15	39-66	2/15	42 median	NA	papillary	NA	NA
160	2017	Shaikh [178]	1	65	NO	24	BILATERAL	biphasic	NO 24	BILATERAL PAINLESS SCROTAL SWELLING
161	2017	An [179]	7	74	NO	NA	NA	biphasic	NA	HYDROCELE
				67	YES	47	NA	biphasic	NO 47	SCROTAL MASS
				58	NO	65	NA	epithelioid	NO 65	SPERMATOCELE
				43	NO	14	NA	epithelioid	14 recurrence	SCROTAL MASS
				47	NO	155	NA	NA	NO 155	HYDROCELE
				85	YES	19	NA	epithelioid	NO 19	HYDROCELE
				71	NO	15	NA	NA	NO 15	HYDROCELE
162	2017	Maheshwari [180]	1	20	NO	16 dead	LEFT	NA	NA	SCROTAL SWELLING
163	2018	Abello [181]	1	80	NO	26	RIGHT	biphasic	24 recurrence	PAINLESS TESTICULAR MASS
164	2018	Trenti [182]	1	40	NO	72 NED	LEFT	tubulo- papillary epithelioid	72 NED	HYDROCELE
165	2018	Zhang [183]	1	65	NO	72 alive	LEFT	NA	72 metastasis	HYDROCELE BILATERAL
	**2018**	**Current cases**	4	75	Occupational foundry worker for 4 years [46]	141 alive	LEFT	tubulo- papillary epithelioid	No	MASS HYDROCELE
				77	Occupational asbestos cement worker for 23 years asbestosis pleural plaques [45]	2 dead	LEFT	tubulo- papillary epithelioid	2 metastasis	MASS
				78	Occupational ship machinist ship then reclaimed for 3 years [58]	40 dead	LEFT	tubulo- papillary epithelioid	cardiopath	MASS
				63	Occupational mason cutting plates eternit trucker for 14 years [41]	3 alive	LEFT	tubulo- papillary epithelioid	Recent case 3 months alive	MASS HYDROCELE

LEGEND: NA not available, NED no evidence of disease, DOD dead of disease, CT computer tomography, MUMP mesothelioma uncertain malignant potential

Watenabe (1994)) [79] and Ascoli (1996) [89] reported two cases of multifocal mesothelioma; the subjects both had occupational exposure as insulators. Individual data were not available in three papers: Spiess (2005) [117], Gemba (2012) [152] and Recabal (2017) [178] which present case series.

Our cases were retrieved from the Apulian malignant mesothelioma register Cor Apulia (Cor-operating centre regional), established in 1993 as a part of the ReNaM-Italian national mesothelioma register. The Apulia mesothelioma register collects data on all incident cases of mesothelioma (pleura, pericardium, peritoneum and tunica vaginalis testis) from 1993 to date.

The regional register according to the national guidelines [185], using a standardized questionnaire and with direct interviews with patients or their relatives, obtained occupational and residential-environmental histories, lifestyle habits and the hobbies of the patients. Similarly, the best evidence of histological diagnosis, follow-up data and vital status of each patient were recorded.

## Results

Since, in 1943, a confusing nomenclature arose, and in 1945 Golden and Ash [18] introduced the term “adenomatoid tumours”, De Klerk and Nime [186] reported in 1975 that, from 1912 to 1975, two hundred three cases of adenomatoid tumours (malignant adenomatoid tumours) of testicular and paratesticular tissues were reported in the English language literature. Therefore, Bisceglia (Bisceglia 2010) [135] reported fewer than 250 cases of testicular and paratesticular mesothelioma, Jankovichova reported approximately 250 cases, and Mrinakova reported approximately 300 cases [165, 171]. All of these cases comprised and could be categorized as MM, WDPM and MUMP or MLMP.

In our review, we found 289 cases of MMTVT (Table 1). The last four cases reported in Table 1 were cases currently found in the Apulia regional registry of the mesothelioma, so the total number of cases reported is here 293.

Among the 289 cases reported here from the literature, the main features are summarized in Table 2.

**Table 2:** 289 MMTVT main features: age at diagnosis, side, histologic type, clinical presentation, duration of follow up, recurrence, asbestos exposure

	Number of cases	%
Age at diagnosis		
1-30	27	9.3
31-40	24	8.3
41-50	32	11.07
51-60	43	14.8
61-70	64	22.1
71-80	48	16.6
81-	17	5.8
NA(not available)	34	11.7
Total	289	100
Laterality		
Right testis	92	31.8
Left testis	104	35.9
Bilateral	6	2.07
Others (spermatic cord, scrotum, epididymis, ecc.)	7	2.4
NA	80	27.6
Total	289	100
Histologic type		
Epithelial	155	53.6
Biphasic	45	15.5
Sarcomatous	5	1.7
NA	84	29.06
Total	289	100
Clinical presentation		
Mass	55	19.03
Hydrocele	84	29.06
Swelling	79	27.3
Others (inguinal hernia, pain, hematoscrotum,ecc.)	15	5.1
NA	56	19.3
Total	289	100
Duration of follow up in months		
2-12	66	22.8
13-36	88	30.04
37-60	27	9.3
61-96	21	7.2
97-132	6	2.07
133-564	7	2.4
NA	74	25.6
Total	289	100
Recurrence		
Metastasis	23	7.9
Multifocal	53	18.3
No	110	38.06
NA	103	35.6
Total	289	100
Asbestos exposure		
Yes	80	27.6
No	88	30.4
NA	121	41.8
Total	289	100

**Fig. 1:**
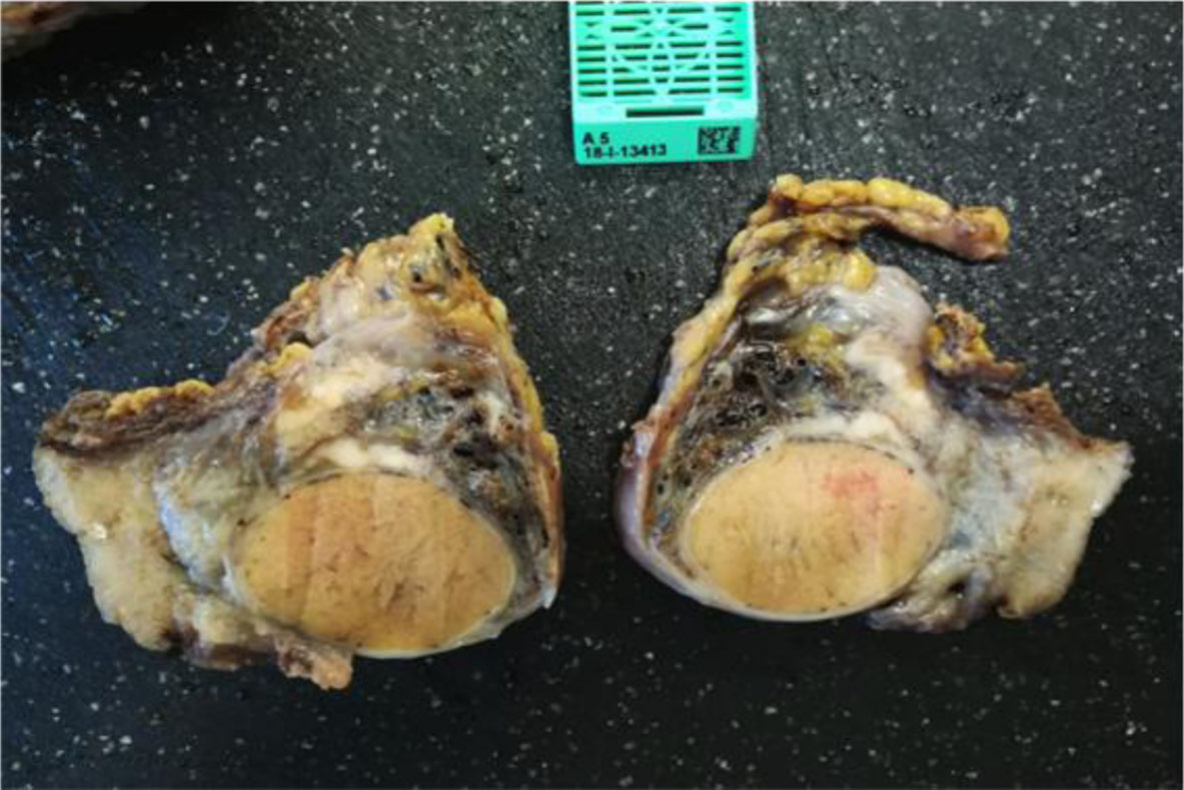
Case number 4 Gross examination, lardaceous superficial thickening of the tunica albuginea

**Fig. 2:**
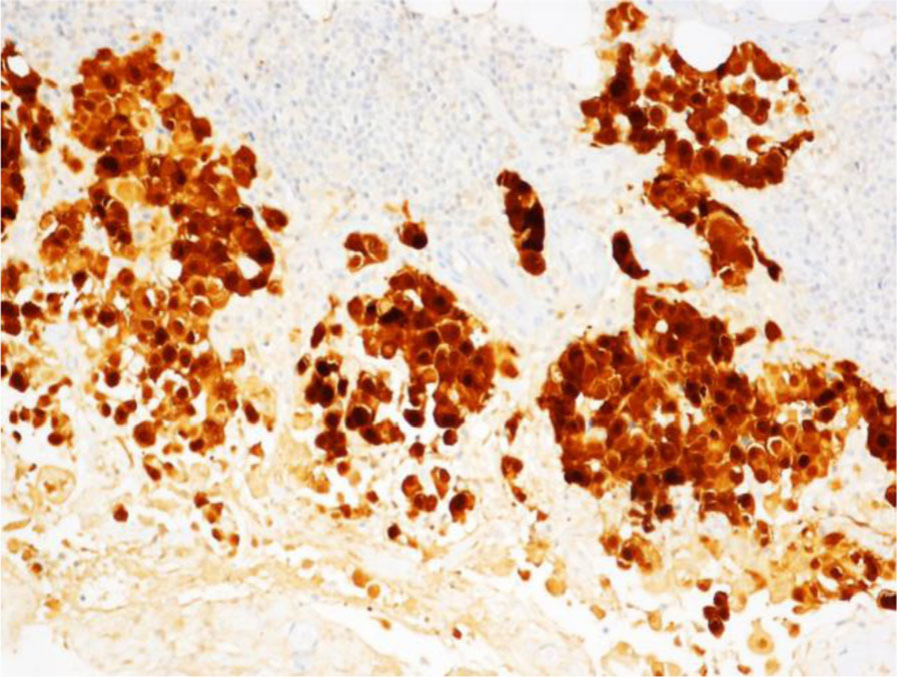
Case number 4. Microscopic examination, pseudopapillary epithelioid neoplastic proliferation wrapping around the testicular parenchyma. Diffuse immunopositivity for calretinin antigen (× 200)

**Fig. 3:**
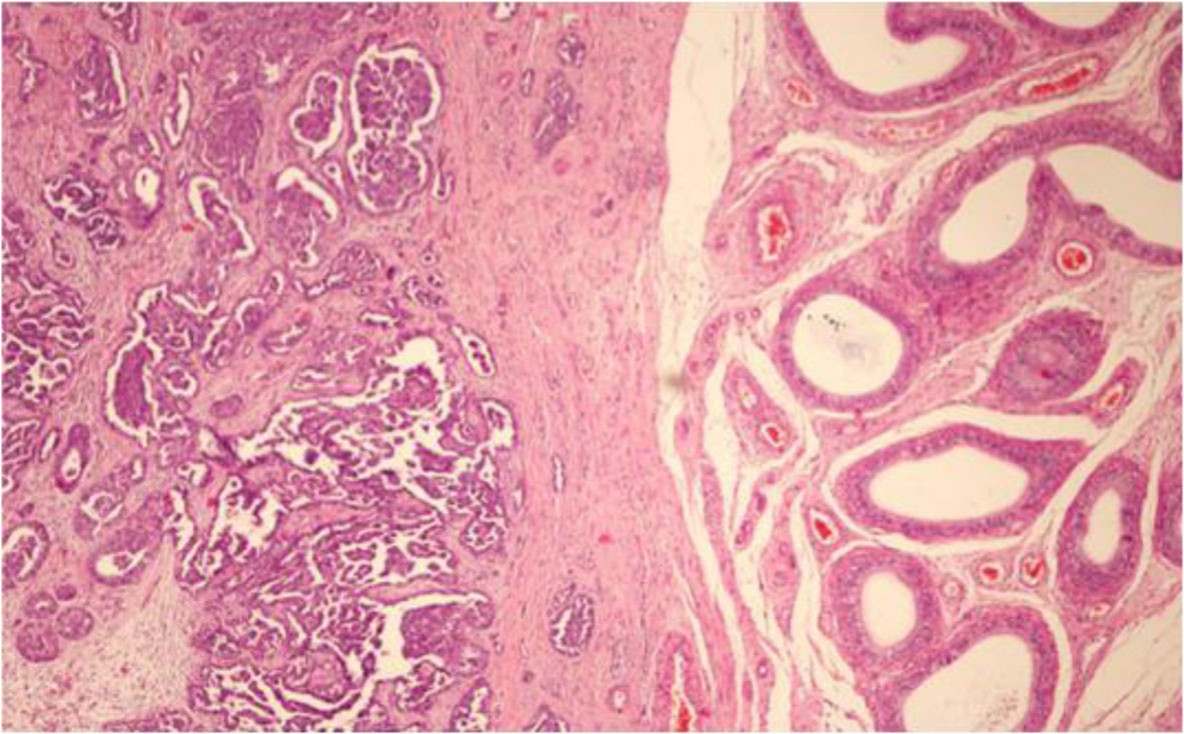
Case number 4. Microscopic examination, pseudopapillary epithelioid neoplastic proliferation wrapping around the testicular parenchyma (H&E, X100)

The characteristics of our cases are reported in Table 1 and summarized in Table 3; the age at diagnosis ranged from 63 to 78 years old, with an average age of 73.2 years old; the clinical onset was a mass, and only two cases also had hydroceles; all of the cases involved the left testicle. All of the patients underwent surgery (orchidectomy), and the histological types were epithelioid. IHC (immunohistochemistry) was always performed with calretinin, HBME1, CK AE1/AE3, EMA positive (Figs. 1, 2, and 3).

**Table 3:** Four MMTVT cases from the Apulia mesothelioma register

Case number	Year of diagnosis	Age years	Clinical diagnosis	Histological diagnosis	IHC	Survival months	Exposure reliable professional	Duration of exposure Years- calendar years	Latency years
1	2006	75	CAT	mm epithelioid with papillary tubule aspects	Calretinine+++, HBME1+++	141	foundry worker	4 (1960-63)	46
2	2009	77	CAT ecocolordoppler	mm epithelioid with papillary and microcystic aspects	Calretinine +++,CK AE1/AE3+++, vimentine+++, WT1 (80%) KI67(8%) nuclear grade 2, IM:3x10HPF	2	asbestos cement worker	21 (1964-85)	45
3	2009	78	CAT ecocolordoppler	mm epithelioid papillary tubule growth pattern solid and focally clear cell presence psammomatous bodies	Calretinine +++, CK AE1/AE3+++,EMA +++, HMBE1 +++, WT1+++ > 25% nuclear grade 2,3 IM:5x10HPF	40	naval machinist	3 (1951-53)	58
4	2018	63	CAT	mm epithelioid papillary (70%) and solid (30%) tubule growth pattern	Calretinine +++,CK AE1/AE3+++,HMBE1 +++, WT1(90%)KI67 10% papillary tubulum component and 40% solid component	3	bricklayer cutting plates eternit and trucker	14 (1977-90)	41

All of our cases were directly interviewed, and asbestos exposure was documented. Two patients had pleural plaques found on computerized axial tomography (CAT) examination. At the last date of follow-up, in September 2018, two patients were alive and two were dead: one died of disease metastasis, and the other died of cardiopathy. The median survival was 46.7 months (range 2-141), the latency period was a mean of 47.5 years (range 41-58), and the duration of asbestos exposure had a mean of 10.5 years (range 2-21).

## Discussion

MMTVT is a rare neoplasm that constitutes 0.3-5% of all mesothelioma cases with a mortality rate of 53% at 2 years following diagnosis [176].

Pathogenesis predisposing factors are described as local trauma, herniorrhaphy, long-term hydrocele or spermatocele [129, 187–189], venereal diseases and ionizing radiation [96, 99].

The tunica vaginalis has a common embryological origin with the visceral pleura, peritoneum and pericardium [187]. Relative to histogenesis in the past, four embryologic hypotheses have been considered: endothelium, epithelial, mesonephric and mesothelial hypotheses [42]. Early writers believed that this tumour had a lymphangiomatous origin because the predominance of labyrinthine channels lined by seemingly flat endothelial cells embedded in a reticular stroma, subsequent microscopic investigations excluded the endothelial origin due to the presence of vacuolated cuboidal and columnar cells. [42].

The mesothelial character was supported by electron microscopic studies [190]. The tunica vaginalis develops from evagination of the peritoneum during fetal life, and it is an embryonic extension of the peritoneal mesothelium, resulting from the descent of the testis through the abdominal wall via the inguinal canal into the scrotum [68]. The epithelial lining of the urogenital tract has mesodermal (mesothelial) origin [191], and the mesothelium has the ability to differentiate into fibroblasts and mesonephric tubular structures, or rather, the mesothelial cells could have a multipotent evolution; they can differentiate in an epithelial or a fibroblastic direction [33, 192, 193]. The mesothelial hypothesis was also corroborated by the occurrence in a patient affected by Maffucci’s syndrome, a mesenchymal disease [50].

To date, there is agreement regarding some of the main features of this disease as shown below; moreover, it is difficult to diagnose preoperatively.

### Symptomatology

MMTVT can be asymptomatic for a long time. Hydrocele, scrotal mass, a lack of pain, inguinal hernia, spermatocele, testicular torsion, previous herniorrhaphy, and post-traumatic injury are all possible clinical manifestations of the disease [147]. Long asymptomatic intervals from initial presentation to clinical recurrence have been reported [54], moreover, MMTVT might mimic epididymitis [147] .

### Diagnosis

Computed tomography, ultrasound, ultrasonography (colour Doppler sonography), and cytological examination of the hydrocele fluid by sonographically guided fine-needle aspiration (FNA) [128, 137, 164] have been performed, although some authors [169, 173] do not agree with these methods due to the low sensitivity of cytology and the potential risk of metastasis, instead using gross pathology images and magnetic resonance imaging.

### Macroscopic appearance gross findings

A firm painless scrotal mass [194], numerous small papillary lesions or multiple nodules studded on the internal surface of the hydrocele sac, diffuse thickening of the tunica vaginalis [195], and a solid coat around the tunica vaginalis with variable features.

### Microscopic appearance findings

Malignant character is demonstrated by the growth pattern, cytological alterations, extensive tissue invasion, and metastases to the lymph nodes; early diagnosis is by cytologic examination of the hydrocele fluid. Nuclear atypia, mitotic activity, with a stroma invasion infiltrative pattern. Cellular nuclear pleomorphism and papillary configuration are signs of lethal potential [52]. An infiltrative pattern of growth with increased cellularity nuclear pleomorphism and high mitotic rate and stromal invasion [105, 195]. Large lymphoid cells with clear or slightly eosinophilic cytoplasm with large strongly atypical polymorphic nuclei and a great number of mitoses; epithelioid features with papillary growth, papillary tubules, and solid growth in invasive foci [196].

### Histologically, it can be of three histologic types

Epithelial (papillary, tubuloalveolar-glandular or solid) [99], fibrosarcomatous or mesenchymal; biphasic; or mixed, associated with the papillary architecture with stromal invasion. Hallmarks of mesothelioma are epithelial cuboidal cells with microvilli, basement membranes, filaments and desmosomes [197].The criteria for malignancy are nuclear pleomorphism, mitotic activity and stromal invasion [13, 198, 199].

### Histochemical-immunohistochemical features

IHC (immunohistochemistry) shows the presence of both cytokeratin and vimentin, suggesting the diagnosis of mesothelioma. Positive staining for cytokeratin, vimentin and Ema (epithelial membrane antigen), with negative staining for Cea carcinoembryonic antigen, Leu–M1, and cytokeratin 20 CKL20. Epithelial membrane antigen and factor VIII are strongly suggestive for the diagnosis of MM; mesothelioma-related markers include calretinin, thrombomodulin, CK5/6 (pleural), WT1 (Wilms tumour antibody), D2-40, CK7 (tunica vaginalis) [120, 162, 188, 198], CD20 +, and calretinin + [196, 200].

### Electron microscopy

The microvilli are elongated and develop complex throughout the tumour; there are well-defined, mature desmosomes through the interdigitating portions of the cytoplasmatic membrane, and numerous cytoplasmatic filaments are observed [104, 151, 188]. In 2009, the International Mesothelioma Interest Group (IMIG) [201]) recommended IHC as the gold standard for the diagnosis of MM, instead of electron microscopy.

### Ultrasonography [103]

The most common sonographic finding is the presence of heterogeneous nodular or papillary masses of the tunica vaginalis associated with a hydrocele or hypoechoic hydrocele with heterogeneous masses of increased echogenicity at the periphery [74, 115, 187, 202]. Lesions are closely related to the tunica vaginalis [196].

### Laterality

Most cases are unilateral on presentation, while only a few cases of bilateral MMTVT have been reported [131]; in the present review, we found only six cases (2.03%) with reported bilateral disease [50, 93, 97, 111, 175, 179].The case reported by Slaysman (1982) [50] occurred in a young man of 20 years old affected by Maffucci syndrome.

Distant spread usually occurs via lymphatics; the retroperitoneal nodes are the most common site of metastasis, while spinal metastasis was described by Mathew (1996) [90] and cutaneous metastasis has also been reported [34, 53, 61, 170].

The differential diagnosis includes mesothelial hyperplasia, adenomatoid tumour, benign papillary mesothelioma, borderline serous papillary tumours, serous carcinomas, carcinoma of the rete testis or epididymis and metastatic adenocarcinoma [26, 99, 188, 203]. Because of potential misdiagnosis, the best evidence for definitive diagnosis requires a panel of HIC markers [145, 198, 203].

The prognosis is poor. While MM of the pleura and peritoneum has an extremely poor prognosis, MMTVT has a better prognosis, but the natural history of this tumour suggests an aggressive behaviour, with a survival rate of less than 50% 2 years after diagnosis [176].Early diagnosis is of great importance for treatment and long-term survival, especially in young men [156, 160].

### Treatment

A multidisciplinary approach of radical orchiectomy and retroperitoneal node dissection is the best choice for cases of this disease. Chemotherapy can be useful for regression of disseminated disease, although to date, because of the rarity of this disease, no statistically significant studies or large series are available to assess the role of adjuvant therapy (chemo- and radiotherapy) [204]. Long-term follow-up over 5 years is needed because late recurrence is not rare and, to date, an aggressive surgical approach is necessary to achieve a cure because of potential late recurrence or metastasis. Many authors have emphasized the importance of considering this tumour in men with scrotal masses and hydroceles [54], even in the absence of asbestos exposure [115, 122, 123]. Lifelong follow-up and management in a multidisciplinary setting are recommended [161, 168, 171].

Similarly, our review, which considered only malignant mesotheliomas in the English literature, as reported by the authors of the examined articles due to the temporal evolution of the histological classification of this pathology, as already noted, does not confirm the total number of cases as reported in previous reviews [149] including approximately 250 cases.

Another limitation of this review is that no best evidence of diagnosis from early articles and no best evidence of asbestos exposure are available.

The histologic prevalent pattern is epithelial (53.6%of all cases), followed by a mixed biphasic pattern in 15.5% and a fibrous sarcomatoid variant in 1.7%. The more frequent age at presentation ranges from 61 to 80 years old (38.7%). Hydrocele was present in 29.06% of the cases described and swelling in 27.3%. Two cases [79, 89] were not primary tumours but of multifocal origin, and the pleura and peritoneum were involved in two patients with heavy exposure to an insulator.

Only 4.4% of cases had a follow-up of over 8 years. Sixty-six patients died of disease progression with an average survival of 24.2 months (range1-76); two cases with a long duration of follow-up died after 132 and 564 months; ultimately, the prognosis remains poor with only rare long-term survivors. The overall recurrence rate (recurrence or metastasis) was 26.2%, predominantly within the first 2 years of follow-up. Both cases reported by Mathew (1996) [90] presented spinal metastasis, and the case reported by Hispan (2016) [170] presented cutaneous metastasis. Finally, in the papers by Spiess (2005) [117], Gemba (2012) [152] and Recabal (2017) [178], no individual data were reported. In previous reviews, a statistically significant correlation was reported between survival with age < 60 years old and organ-confined disease at diagnosis [74, 202]. Assessment of prognostic parameters revealed a significant correlation of the patient’s age with survival [96]. Radical inguinal orchiectomy might contribute to a better prognosis [112]. Due to the possibility of late tumour recurrence reported in 2.7% [96], lifelong follow-up can be recommended and should be offered to the patient because of the metastatic potential of the tumour; in fact, recurrence can occur as late as 15 years postoperatively [123, 205].

Regarding risk factors, the only causal factor so far ascertained is asbestos exposure, and exposure to different asbestos-containing materials is the only well-documented risk factor [87, 96], as stated by IARC (international agency on cancer research) (2012) [1], although information about exposure might not always have been adequate. Nevertheless, there are authors who do not agree with the absence, until today, of analytical case-control epidemiologic studies to test this relationship [189].

Asbestos is an ascertained carcinogen [1] in the development of mesotheliomas. It is necessary to bear in mind that it is ubiquitous not only in the workplace but also in the general environment [206]. The first study reporting an MMTVT case, diagnosed in 1969, with asbestos exposure was published in 1976 by Fligiei and Kaneko [38] in a pipe insulator exposed for 40 years. In the same year, Pizzolato and Lamberty [40] reported a case in a sugar refinery worker. Since the first case of MMTVT described in 1976 by Fligiel and Kaneko (Fligiel 1976 [38], it has been supposed that the asbestos fibres from the lung can reach the tunica vaginalis by a lymphatic or bloodstream route [207, 208]. Mirabella (1991) [209], in his review of the literature, reported eleven cases with occupational asbestos exposure. In the review by Jones (1995) [87] of a total of 63 cases, 48% had histories of asbestos exposure, while in Mensi’s report (2012) [149], 61% of cases had asbestos exposure.

Overall, asbestos exposure was investigated only in 58% of all cases reported in this review, while in 41.8%, these data were not available. Notably, in many reports, there was no anamnestic reconstruction of any asbestos exposure.

A history of direct occupational, environmental or family asbestos exposure is found in 27.6% of these cases. Among these cases (80 cases) 12.5% reported generic occupational exposure the others 87.5% have a documented history of asbestos exposure. Among the latter there are insulators, dock workers, steel industry workers, farmers, shipyard workers and other different occupations in sectors known to involve asbestos exposure. To be noted there are four cases with environmental exposure, six with household, family or hobby exposure and five cases with or without declared exposure but with pleural plaques or asbestos bodies.

The duration of asbestos exposure is recorded in 108 articles of the 165 reviewed (65.45%). In these articles 50 ascertained the exposure (30.30%) while in 58 articles it was excluded (35.15%).

The duration of exposure is between 4 and 50 years, for occupational exposures only the range is 4-40 years. For the new employment cases presented here the range is 3-23 years.

The true incidence of asbestos exposure in these reported MMTVT cases is underestimated because of insufficient information, especially for the earlier cases and case series described until the beginning of the 2000s, when the scientific community became aware of the risk factors for this disease represented by asbestos exposure [158]. Similarly, because of the long latency period, even over decades, poor patient recall in the reconstruction of asbestos exposure and occupational histories or the patient being unaware of using materials containing asbestos [171], until now, the quality of these data was quite unclear, which might have caused the majority of MMTVT cases to date appearing to be idiopathic, and there is no accurate assessment of asbestos exposure association. However, latency in Antman’s (1984)) [54] case series ranged from 16 to 40 years. The higher incidence of MMTVT among older patients is related to longer exposure to asbestos with a latency range of 10-40 years. A positive history of asbestos exposure or asbestos-containing materials constitutes a risk for the development of an MMTVT and should be monitored [123].

The Apulia mesothelioma registry recorded 4 cases of MMTVT from 1993 to 2018, accounting for 0.3% of all MM cases reported in the regional register during this period. This percentage is consistent with the national Renam data (0.28%) from the national Italian mesothelioma registry [2]. The age at diagnosis was an average of 73 years old, and the mean survival (46 months) was consistent with that reported in the literature [188]. The family histories and clinical-medical histories of the patients were unremarkable. None of our patients underwent chemotherapy or radiotherapy cycles after orchidectomy. The Renam data [2] showed that more than 59% of MMTVT cases had asbestos exposure. Our four cases, all with occupational exposure, had a latency of 47 years and an exposure length of 10.5 years, and these data are concordant with the descriptions in the literature of the aetiological role of asbestos in the pathogenesis of MM [3, 13, 206]. The accurate diagnosis of primary malignant MMTVT and occupational anamnesis are helpful for medicolegal compensation considerations, especially for the cases associated with asbestos exposure [13]. The case described here was referred to the Italian workers’ compensation authority (Inail - National Insurance Institution for Occupational Accidents).

Recently, many studies have demonstrated molecular changes in MM with multiple chromosomal alterations [184, 210–214]. Chromosomal abnormalities in cases of MMTVT were described for the first time by Serio (Serio 2016) [174]in two cases with comparative genomic hybridization (CGH) findings. The two cases showed several gains and losses, in particular, identical lost regions at 1p13.3 → q21.1; 19q13.42; 21q22.2; and 22q12.2 (tumour suppressor gene NF2). Jean (Jean 2012) [215] hypothesized that NF2 regulates cell growth function, and its inactivation could be related to tumour progression and patient survival. We are deepening the study of these new cases, all with ascertained exposure to asbestos, to understand whether there are specific DNA copy number changes in MMTVT and investigating the relative genes involved to define whether they are or are not the same as those reported in pleural MM, particularly in relation to asbestos exposure, and whether they might be useful in elucidating tumorigenesis and predicting prognosis.

## Conclusions

Although this systematic review shows that only 27.6% of the cases reported in this long period of time (1943-2018) had asbestos exposure must be underlined that in 41.8% of the cases in the literature exposure to asbestos is not investigated. In our opinion, to establish a broad consensus on the causal relationship between asbestos and MMTVT in the scientific community, we will need to analyze these relationships with analytical epidemiological studies. A case control study on the data from the national mesothelioma registry is under way in Italy, together with molecular epidemiological studies.

## Data Availability

The dataset and articles used and analyzed during the study are available from the corresponding author on reasonable request.

## Abbreviations

CAT: Computerized axial tomography
CGH: Comparative genomic hybridization
COR: Operating centre regional
FNA: Fine needle aspiration
IARC: International agency on cancer research
IHC: Immunohistochemistry
IMIG: International mesothelioma interest group
MLMP: Mesothelioma low malignant potential
MM: Malignant mesotelioma
MMTVT: Malignant mesothelioma tunica vaginalis testis
MUMP: Mesothelioma uncertain malignant potential
NA: Not available
ReNaM: National mesothelioma register
WDPM: Well differentiated papillary mesotheloma
WHO: World Health Organization
